# Supplementary material for: Multi-Omic Identification of Venom Proteins Collected from Artificial Hosts of a Parasitoid Wasp
Source: Toxins (Basel). 2023 Jun 3;15(6):377. doi: 10.3390/toxins15060377 (PMC10304110; doi:10.3390/toxins15060377)
Supplement: Supplementary file 1 [file toxins-15-00377-s001.zip › Toxins_Supplementary Figures.pdf]

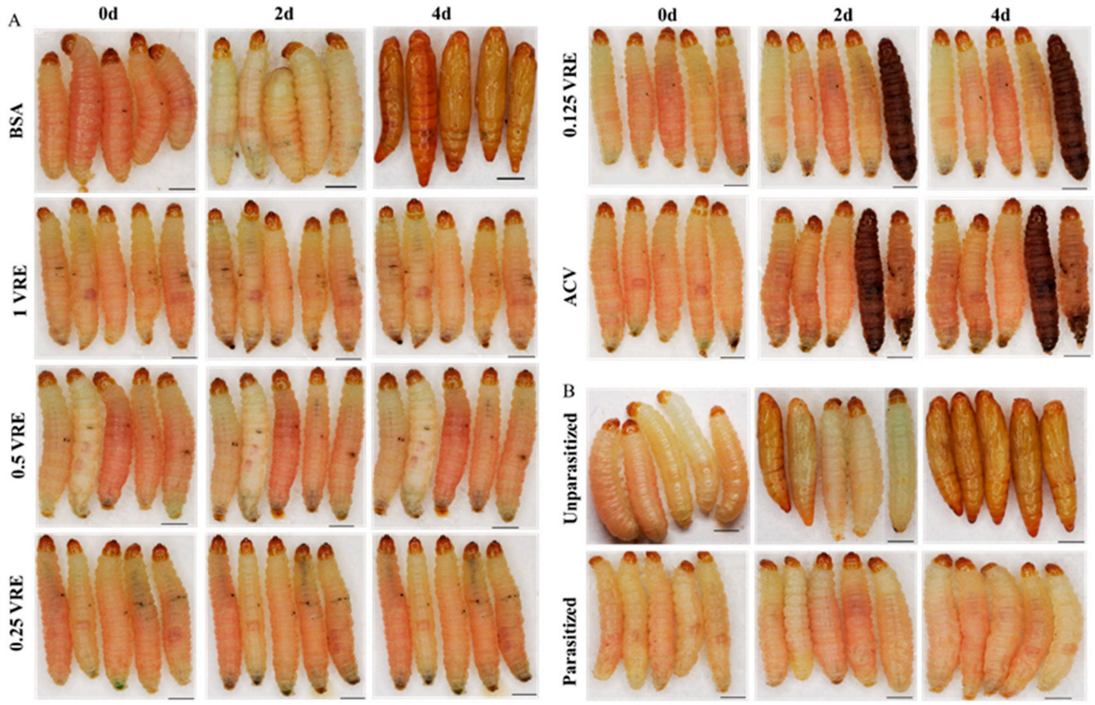

Figure S1 Effect of the venom proteins on hosts. (A)Effect of different equivalents of coarse venom and artificially collected venom proteins on hosts. VRE, venom reservoir equivalent; ACV, artificial collection venom from mimicked hosts; BSA, bovine serum albumin. (B) The unparasitized and parasitized hosts.

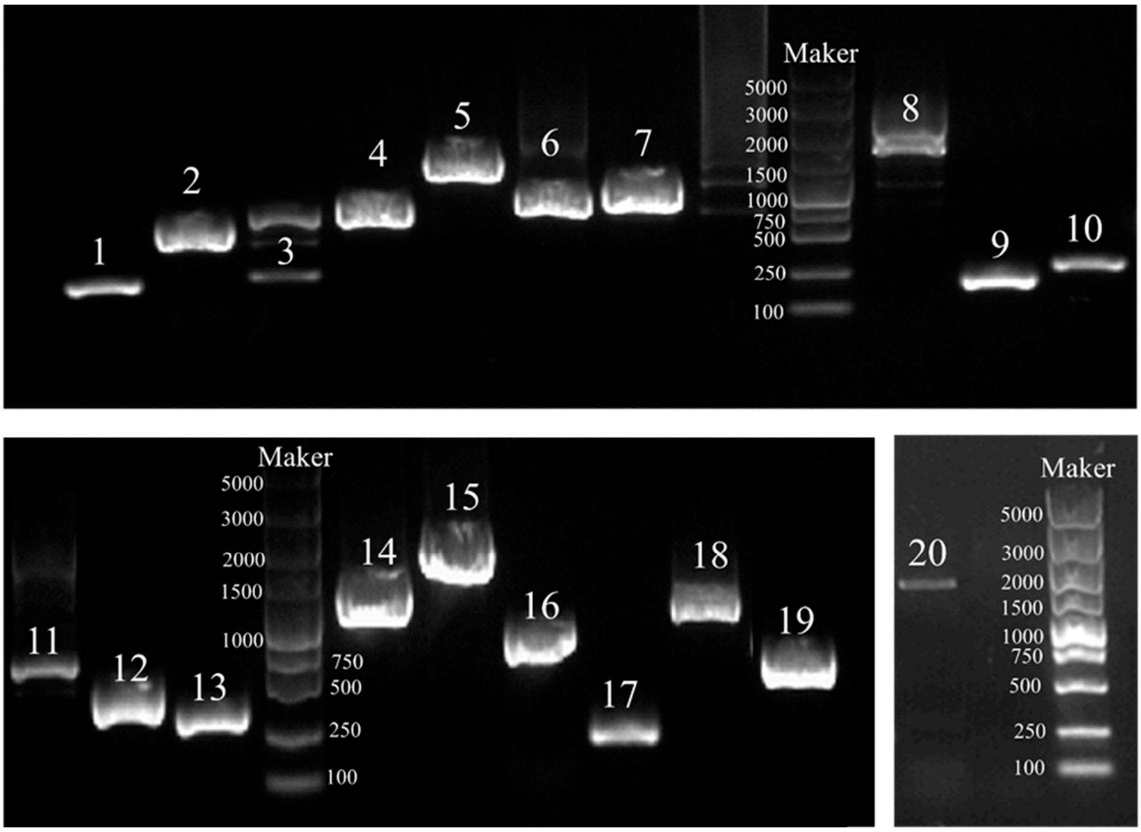

Figure S2 The gel electrophoresis of 20 selected ACV PCR product fragments. The serial numbers were marked above the target strip of each corridor. 1, Hheb062860.1; 2, Hheb016100.1; 3, Hheb070790.1; 4, Hheb002640.1; 5, Hheb104600.1; 6, Hheb120010.1; 7, Hheb035210.1; 8, Hheb057830.1; 9, Hheb020350.1; 10, Hheb056680.1; 11, Hheb015940.1; 12, Hheb000690.1; 13, Hheb084530.1; 14, Hheb026410.1; 15, Hheb024590.1; 16, Hheb016660.1; 17, Hheb064260.1; 18, Hheb106520.1; 19, Hheb005270.1; 20, Hheb093810.1. ACV, artificial collection venom from mimicked hosts; PCR, polymerase chain reaction.

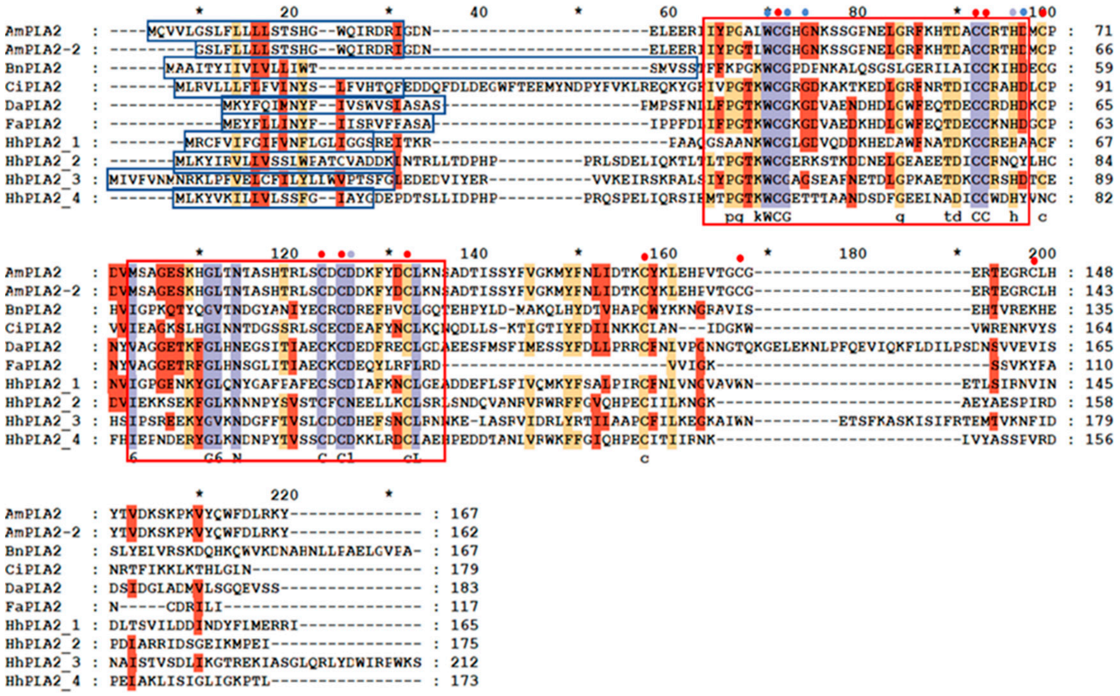

Figure S3 Multiple sequence comparison of 8 phospholipase A2-like (PLA2) proteins. Blue boxes indicate signal peptides. Red boxes indicate conserved structural domains. The dashes represent gaps introduced to maintain alignment. Red dots indicated conserved cysteine residues, blue dots indicated Ca^{2+} binding sites and purple dots indicate active sites. AmPLA2, AFI40558.1; AmPLA2-2, CAA34681.1; CiPLA2, XP_034943085.1; DaPLA2, XP_015109152.1; FaPLA2, XP_011309464.1; HhPLA2_1, Hheb079670.1[2,3]; HhPLA2_2, Hheb088590.1[1,2,3]; HhPLA2_3, Hheb112070.1[2]; HhPLA2_4, Hheb088580.1[1].
